# Therapeutic Approach in Language and Cognitive Skills in Premature Twins with ASD: Case Report

**DOI:** 10.3390/bs15111587

**Published:** 2025-11-19

**Authors:** Alejandro Cano-Villagrasa, Fatma Ben-Mansour, Miguel López-Zamora, Isabel López-Chicheri

**Affiliations:** 1 Departamento de Logopedia, Facultad de Ciencias de la Salud, Universidad Internacional de Valencia (VIU), 46002 Valencia, Spain; 2 Departamento de Psicología, Facultad de Ciencias de la Salud, Universidad Europea de Canarias, La Orotava, 38300 Tenerife, Spain; 3 Departamento de Psicología, UCAM Universidad Católica de Murcia, 30107 Murcia, Spain; fbenmansour@alu.ucam.edu (F.B.-M.); ilchicheri@ucam.edu (I.L.-C.); 4 Departamento de Psicología Evolutiva y de la Educación, Facultad de Psicología y Logopedia, Universidad de Málaga, 29071 Málaga, Spain

**Keywords:** prematurity, ASD, language, communication, cognition

## Abstract

Prematurity and autism spectrum disorder (ASD) are risk factors for alterations in language development. Their coexistence, frequent in twin pregnancies, may result in atypical communicative profiles that require specific interventions. This case report analyzed the linguistic, cognitive, and socioemotional development of two premature twins with ASD, relating the results to the therapeutic strategies applied. Standardized tests were applied to measure cognitive, linguistic, adaptive, and socioemotional development. The intervention combined the TEACCH, ABA, DIR/Floortime, and Hanen—More Than Words models. Both children showed significant impairments in communication, executive functions, and autonomy, with differentiated clinical profiles. Individualized interventions favored advances in functional language, emotional regulation, and routines, although challenges in language generalization and pragmatics persisted. The combination of prematurity and ASD creates complex challenges that require individualized therapeutic approaches. Early and intensive intervention, based on structured and relational approaches, is useful to promote functional and communicative development.

## 1. Introduction

Prematurity is defined as birth before 37 weeks gestation and affects approximately 10% of births worldwide (March of Dimes, 2020). This condition is associated with an increased risk of alterations in brain connectivity, which can significantly impact neurological, cognitive, and behavioral development (Rogers et al., 2018). Research has pointed to structural and functional differences in critical regions such as the cerebellum, corpus callosum, and arcuate fasciculus, structures critical for language processing and social interaction (Vandormael et al., 2019).

One of the most prevalent neurodevelopmental disorders in preterm children is autism spectrum disorder (ASD). Longitudinal studies have shown that preterm children are up to 10 to 12 times more likely to be diagnosed with ASD compared to the general population (Ouss-Ryngaert et al., 2012; Allen et al., 2020). Communication difficulties, widely documented in children with ASD, include alterations in vocabulary development, morphosyntax, lexical-semantic ability, and pragmatics, which significantly affect their social interaction and adaptation (Cano-Villagrasa et al., 2023, 2024). In this context, both prematurity and ASD are independent risk factors for language impairment, but their combination could exacerbate communicative deficits (Harel-Gadassi et al., 2018; Hariningtyas et al., 2022).

To better delineate the contribution of prematurity versus ASD, it is important to note that preterm children without ASD often show difficulties primarily related to attention, executive functioning, and expressive language delay, but they typically preserve pragmatic and social reciprocity skills (Rogers et al., 2018). In contrast, children born at term with ASD present a wide heterogeneity of cognitive and linguistic profiles, ranging from minimally verbal cases to those with high verbal ability but marked pragmatic difficulties (Lord et al., 2020). Understanding these distinctions is essential to interpret the developmental patterns of premature children with ASD, as their communicative and socioemotional profiles are shaped by the interaction of both risk factors rather than by ASD alone.

Language development in preterm infants has been extensively studied, with evidence indicating that up to 73.3% of these children have language deficits, with a 3.57-fold increased risk compared to those born at term (Maggiolo et al., 2014; Hariningtyas et al., 2022). Previous research has documented delays in vocabulary, grammatical structure, and contextual language use in children born extremely preterm (Foster-Cohen et al., 2007). In this regard, Joseph et al. (2023) found that these children experience significant delays in word production, with a mean onset later than 24 months, and in sentence consolidation, which occurs after 33 months. In addition, Sansavini et al. (2019) reported that preterm children with ASD risk or language delay present lower use of deictic gestures and deficits in social interaction compared to those born at term.

It has been suggested that children with ASD born preterm may have a different phenotype compared to those born at term. Specifically, preterm infants with ASD tend to show less presence of repetitive behaviors, but increased difficulties in language and communication development (Joseph et al., 2023; Luu et al., 2020). This phenotypic difference suggests that the combination of prematurity and ASD may result in distinct clinical profiles that require specific evaluation and intervention strategies.

Within this context, twin pregnancies represent an additional risk factor. Although they constitute 3.2% of live births, they account for up to 20% of preterm pregnancies. Of these, 60% occur before 37 weeks and 10.7% before 32 weeks of gestation (Roman et al., 2022). Prematurity is six times more frequent in twins, leading to lower birth weight and increased exposure to prematurity-related diseases and long-term complications (Giuffrè et al., 2012). In addition, studies point to a high concordance of ASD in monozygotic twins, reaching up to 88% (Rosenberg et al., 2009), thus reinforcing the shared genetic and environmental influence. Moreover, premature twins usually present atypical language development and neurological alterations that may interfere with language acquisition (Souza et al., 2019).

Despite the growing interest in the relationship between prematurity, ASD, and language development, studies specifically analyzing this intersection are limited. Most research addresses prematurity and ASD as independent factors, without considering how their combination may impact language development.

Therefore, this case report aims to analyze language development in two premature twins diagnosed with ASD, integrating linguistic, communicative, cognitive, and socioemotional dimensions. By linking these findings with the multimodal therapeutic approaches implemented, this study seeks to provide a nuanced understanding of how combined models such as ABA, TEACCH, DIR/Floortime, and Hanen can support language and social–emotional growth in premature children with ASD.

## 2. Description of the Cases

### 2.1. Perinatal and Family History

The twins JR and SR were born on 19 October 2018, making them currently 6 years old. The pregnancy was obtained by artificial insemination and controlled without significant incidences. The mother, diagnosed with juvenile dermatopolymyositis with chondrocalcinosis, had a high-risk pregnancy, in addition to presenting gestational diabetes. The pregnancy was carried to 36 + 5 weeks and ended with a programmed cesarean section at Hospital La Fe. Both children were born with an appropriate weight for their gestational age: JR with 2240 g and SR with 2810 g.

Neonatal complications were recorded; JR required hospital admission at 56 days of life for an episode of cyanosis, diagnosed as suspected apnea. In addition, a suspected intolerance to cows’ milk protein was observed, which led to a follow-up in gastroenterology. SR, on the other side, presented mild plagiocephaly in his first months of life.

Regarding family history, there are no cases of neurodevelopmental disorders in the maternal or paternal bloodline, although there is a history of epilepsy in the maternal sibling. The father suffers from hypercholesterolemia and the mother from dermatopolymyositis.

Currently, both children attend CEIP Vicente Gaos (Valencia) in mainstream classrooms, receiving additional support from Therapeutic Pedagogy (PT), Hearing and Language (AL), and Special Education (EEE).

### 2.2. Development History

Since the first months of life, JR showed signs of difficulties in language development and social communication. He was referred at 18 months by his pediatrician to neuropediatrics due to a delay in language acquisition and a regression in communicative intention, being diagnosed with grade 3 ASD. At 2 years of age, he was diagnosed with periodic fever associated with PFAPA and treated with colchicine. In addition, he presented restricted feeding, double incontinence, impulsivity, and difficulties in following instructions, reaching risky situations such as running into the road without control. His sleep is irregular with frequent awakenings.

SR, on the other hand, was evaluated at 23 months after a preventive evaluation prompted by her brother’s diagnosis. She showed fewer signs of early development, but her evolution showed difficulties in social communication and a tendency to become self-absorbed. In September 2020, she was diagnosed with ASD grade 1. SR showed cognitive rigidity and difficulties in autonomy in everyday situations.

## 3. Method

### 3.1. Study Design

This paper corresponds to a clinical case report of two patients with ASD and prematurity, which aims to analyze the profile of language development in two premature twins diagnosed with this disorder.

### 3.2. Participants

The participants of this study are twins born on 19 October 2018 by scheduled cesarean section at 36 + 5 weeks of gestation. Both have been under medical and therapeutic follow-up since early ages, after being diagnosed with ASD. JR was diagnosed with ASD grade 3, while SR with ASD grade 1. The assessment and follow-up have been carried out by a multidisciplinary team that includes neuropediatricians, speech therapists, psychologists and professionals from the educational field. Additionally, a parental interview was conducted to obtain detailed family, perinatal, and early developmental history, which informed both assessment and intervention planning.

### 3.3. Instruments

The participants were assessed using a series of standardized tests and clinical instruments selected to provide a comprehensive profile of cognitive, linguistic, adaptive, and socioemotional skills relevant to the study’s objectives:

(a) **Merrill-Palmer scales development (Dempsey et al., 2020):** The Global Development Index provides a broad measure of development, while the Adaptive Behavior Index evaluates functional skills in daily life. Internal consistency ranges from 0.85 to 0.94. It was chosen for its ability to capture global developmental patterns and adaptive functioning relevant to early intervention planning. Subtests include cognitive, motor, language, socioemotional, and adaptive domains.
(b) **The Adaptive Behavior Assessment System (ABAS-II; Harrison et al., 2013):** Assesses adaptive behavior from birth to 89 years. The Global Adaptive Composite (GAC) shows very high internal consistency (0.97–0.99) and high reliability for adaptive domains (0.91–0.98). It evaluates functional independence across conceptual, social, and practical domains, providing relevant information on real-life skills for intervention targeting.
(c) **The Behavior Rating Inventory of Executive Function (BRIEF-2; Bausela-Herreras & Luque-Cuenca, 2017):** Evaluates observable executive functions in children 5–18 years. Internal consistency coefficients range from 0.80 to 0.98. Selected to measure attention, working memory, planning, and self-regulation in everyday contexts.
(d) **Neuropsychological assessment services (Neuro-kid; Portellano Pérez et al., 2021):** Screens global development in children 3–7 years, assessing essential cognitive domains for evolutionary development.
(e) **Developmental Profile-3 (DP-3; Alpern, 2018):** Rapidly assesses cognition, motor skills, socioemotional development, communication, and adaptive behavior in children. Internal consistency ranges from 0.89 to 0.97. Provides a global index of child development.
(f) **Child and Adolescent Assessment System (SENA; Rodríguez Camón, 2018):** Detects disorders and difficulties that occur at various stages of development, from age 3 up to adulthood. Internal consistency coefficients range from 0.77 to 0.96. The tool has been developed to address the challenges most commonly encountered at each stage of development.
(g) **The Clinical Evaluation of Language Fundamentals—5 (CELF-5; Wiig et al., 2018):** Individually administered assessment of language in children aged 5–15 years. Internal consistency ranges from 0.75 to 0.98. Provides detailed evaluation of expressive language such as lexis, morphosyntax, semantics, and pragmatics, as well as receptive language.
(h) **The Illinois Test of Psycholinguistic Aptitudes (ITPA; Kirk et al., 2009):** Evaluates linguistic processing and communication skills in children aged 3–10 years. Internal consistency ranges from 0.80 to 0.92. Used to identify strengths and weaknesses in language processing relevant to the intervention’s design.

### 3.4. Procedure

#### 3.4.1. Assessment Procedure

**Figure 1:**
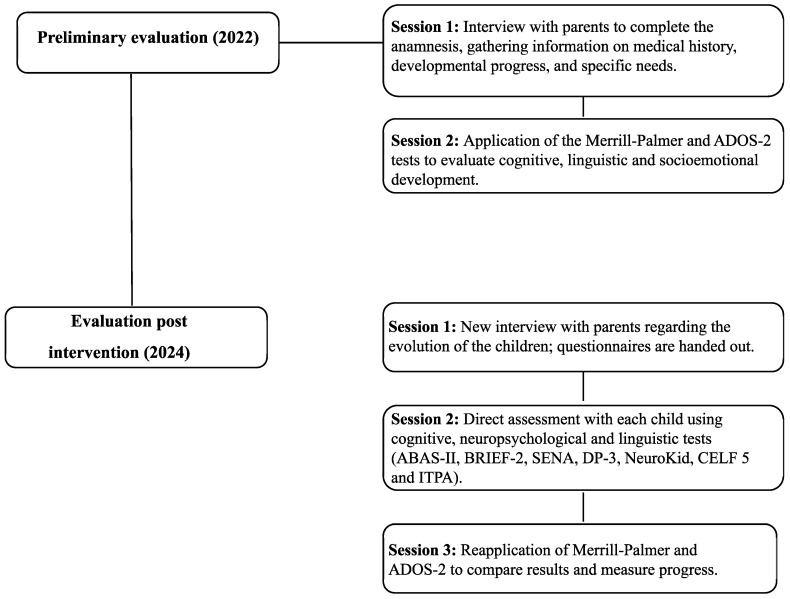
Flowchart of evaluations.

The assessment was conducted in two phases: pre-intervention (2022) and post-intervention (2024) (Figure 1). Each phase included structured sessions designed to capture the cognitive, linguistic, adaptive, and socioemotional development of the twins.

Pre-intervention (2022, 2 sessions on consecutive days):

- Session 1: Parent interview to obtain family history, perinatal and developmental information, and anamnesis. This session ensured comprehensive background knowledge to contextualize the twins’ developmental profiles.
- Session 2: Direct assessment of each child using Merrill-Palmer Developmental Scales. These instruments were applied pre-intervention to establish baseline profiles in cognitive, linguistic, and socioemotional domains, allowing for subsequent comparison with post-intervention outcomes.

Post-intervention (2024, 3 sessions over 1–2 weeks):

- Session 1: Follow-up parent interview focusing on developmental progress and intervention effects.
- Session 2: Direct evaluation using ABAS-II, BRIEF-2, SENA, Neuro-Kid, DP-3, CELF-5, and ITPA. These instruments were chosen to provide a detailed profile of cognitive, linguistic, adaptive, and socioemotional outcomes, reflecting changes potentially attributable to the intervention.
- Session 3: Re-administration of Merrill-Palmer to allow for a direct comparison between pre- and post-intervention performance and assess longitudinal changes.

All sessions were conducted in structured environments adapted to each child’s needs, ensuring optimal assessment conditions. Parents were actively involved, providing consent and additional information to enhance the ecological validity and accuracy of the results.

**Rationale for instrument selection:** Merrill-Palmer was administered both pre- and post-intervention to provide comparable baseline and follow-up measures across cognitive, linguistic, and socioemotional domains. Direct assessments (ABAS-II, BRIEF-2, SENA, Neuro-Kid, DP-3, CELF-5, and ITPA) were used post-intervention to capture the specific skills targeted by the intervention and to evaluate changes across domains.

#### 3.4.2. Intervention Procedure

The intervention was designed to consider the individual and shared needs of the twins, JR and SR, respecting their developmental differences and avoiding treating them as a homogeneous unit. The intervention consisted of approximately 70–80 individual sessions per twin, each lasting 45 min, conducted over a period of around 20 months. Both children received one-to-one sessions led by the same licensed speech and language therapist trained in all four intervention models. A flexible integration of evidence-based frameworks was applied: the TEACCH program (Schopler et al., 1995), Applied Behavior Analysis (ABA) (Cooper et al., 2019), the DIR/Floortime approach (Nowakowski-Sims & Gregan, 2017), and the Hanen—More Than Words program (Sussman, 2012). This integration followed a semi-structured format, adapting manualized components from each model while maintaining fidelity to their core principles.

The intervention also included systematic parent coaching sessions every 6–8 weeks to ensure the generalization of the therapeutic goals at home and in natural environments.

This multimodal design allowed us to address cognitive, linguistic, and socioemotional aspects in a coordinated way, promoting family participation and the transfer of learning to everyday contexts.

##### Cognitive Intervention

The main objective of the cognitive intervention was to strengthen executive functions (sustained attention, cognitive flexibility, working memory, logical reasoning, and planning), simultaneously promoting emotional self-regulation and the ability to adapt to new situations. For this purpose, a personalized intervention program was designed that integrated different methodological approaches and strategies adapted to the individual characteristics of each twin:

1. ABA Model: Structured tasks using forward and backward chaining were applied with immediate positive reinforcement (verbal praise, tokens, or extra playtime). Examples included simple problem-solving, sustained attention activities (e.g., object search and image matching), and adaptive behavior routines to promote autonomy and tolerance to changes.
2. TEACCH Method: The cognitive environment was structured with visual materials to help the understanding of daily and school activities, promote autonomy, and reduce anxiety. Visual schedules, independent task folders, and manipulative materials were used to promote predictability, organization, and independence.
3. DIR/Floortime approach: It allowed us to work on cognitive development in motivating and meaningful play contexts, favoring problem solving in a natural way and linked to the individual interests of each child. This facilitated the integration of cognitive functions with the emotional and social dimensions, fundamental aspects for the development of mental flexibility and symbolic thinking. Activities included guided play following children’s interests, the creation of “communication circles”, and symbolic play to encourage flexible thinking and emotional regulation.

The combination of these methods favored not only the development of basic and complex cognitive skills, but also their functional integration with the emotional, social, and adaptive components of the twins. Thus, a holistic approach was achieved that favored gradual progress in self-regulation, planning, and the flexible resolution of situations in different contexts, promoting learning that is applicable to everyday contexts.

##### Linguistic Intervention

The linguistic intervention focused on improving comprehension and verbal expression, including phonological, semantic, morphosyntactic, and pragmatic aspects.

1. ABA model: Applied verbal shaping, echoic training, and reinforcement techniques. Structured tasks targeted naming, categorization, sentence construction, and comprehension through visual prompts.
2. TEACCH: Provided visual and routine-based supports to aid understanding, such as communication boards, “First–Then” routines, and story sequencing.
3. DIR/Floortime: Promoted the development of language in its functional and social dimension, addressing pragmatic aspects, the use of gestures, non-verbal language and symbolic functions, in emotionally meaningful play contexts. Symbolic games, role-play, and turn-taking activities enhanced intentional communication and social reciprocity.
4. The Hannen program involved the parents, teaching them language stimulation strategies through guided sessions, home recordings, and joint analysis of interactions. This allowed for a reinforcement of the achievements of the therapeutic approach in daily life and for the generation of an environment rich in communicative opportunities.

Thanks to the combination of these models, it was possible to improve communicative initiative, increase verbal content, and promote the generalization of functional language in both the therapeutic and family environments.

##### Socioemotional Focus

Socioemotional aspects were targeted through co-regulation activities, emotional labeling, and social routines integrated across all sessions. DIR/Floortime strategies emphasized emotional attunement and shared affect, while ABA and TEACCH approaches supported behavioral regulation through structure and predictability. The Hanen program reinforced these components within daily family interactions.

## 4. Results

The following results summarize the evaluations carried out on JR and SR, organized in three domains: maturational profile, cognitive profile, and linguistic profile. Data from the pre-intervention (2022) and post-intervention (2024) are presented when available. Interpretations correspond to post-intervention data, with pre-intervention scores included for comparison. For clarity, abbreviations used in the tables are as follows: PT = Mental Age (months), PC = Percentile, and EE = Equivalent Age (months). Normative data are presented when available; mean ± SD values were not provided for all measures.

### 4.1. Development and Maturational Profile

The maturational profile was assessed with Merrill-Palmer (MP), DP-3, ABAS-II, BRIEF-2, and SENA, capturing global development, adaptive behavior, socioemotional skills, and autonomy. The results are presented below, organized by patient, with their respective tables and qualitative analysis of each area evaluated.

- **PATIENT 1—JR**

JR initially showed overall development within an average range. However, he presented significant difficulties with limited social interactions, problems differentiating between familiar people and strangers, and restricted responses to affective signals. In terms of adaptive behavior, significant challenges in personal autonomy were noted, reflecting problems with activities of daily life such as toileting and personal safety. At post-intervention, improvements in adaptive behavior were observed, but scores remained below normative expectations. ABAS-II, BRIEF-2, and DP-3 scores confirmed the need for ongoing support. The SENA profile revealed high levels of anxiety, social isolation, and difficulties with impulsivity and emotional regulation (Table 1).

Intervention response: JR participated actively in sessions, showing gradual improvement in adaptive routines and engagement, though expressive language challenges persisted.

- **PATIENT 2—SR**

SR presented a maturational profile more adjusted to his age, with scores within the average range in general development, but showed difficulties in the distinction between familiar people and strangers, and her interactions with other children were occasional and not very sustained. In terms of adaptive behavior and self-care, she presented limitations in personal autonomy, with difficulties in expressing discomfort and carrying out familiar routines. Post-intervention, difficulties in autonomy and emotion regulation persisted, with moderate progress in the 2024 evaluation. ABAS-II and SENA confirmed difficulties in adaptive behavior and emotional regulation, particularly in personal autonomy, social reciprocity, and emotion management (Table 2).

Intervention response: SR participated consistently in sessions, showing improved compliance with routines and better emotional regulation, especially in structured tasks.

### 4.2. Cognitive Development and Profile

The evaluation of the cognitive profile was performed with the Merrill-Palmer, DP-3, BRIEF-2, ITPA, NeuroKid, and SENA Profile, assessing areas such as cognition, memory, executive functions, processing speed, and visuoperception.

- **PATIENT 1—JR**

JR presented general cognitive development within the average range, with adequate scores in areas such as memory and processing speed. Despite these strengths, cognitive rigidity and difficulties in self-regulation were observed, especially in tasks involving adaptation to change or problem solving. Post-intervention, executive function deficits persisted in flexibility, verbal reasoning, verbal memory, and planning (Table 3).

Intervention response: JR engaged actively in cognitive tasks, with gains in problem-solving and structured tasks, though executive difficulties persisted.

- **PATIENT 2—SR**

SR presented with solid general cognitive development, with adequate scores in memory and processing speed. Post-intervention, difficulties remained in attention, working memory, planning, organization, and cognitive flexibility. NeuroKid results indicated adequate visuoperception and executive function (Table 4).

Intervention response: SR participated actively in cognitive tasks, showing moderate improvement in attention, working memory, and planning, though difficulties in flexibility and verbal reasoning persisted.

### 4.3. Linguistic Development and Profile

Language assessment included Merrill-Palmer, DP-3, CELF-5, ITPA, and NeuroKid tests. These tools allow for the identification of the level of verbal comprehension and expression, pragmatic skills, morphosyntactic structure, functional language use, and phonological skills.

- **PATIENT 1—JR**

JR showed a notable weakness in expressive language, with limited sentence production and difficulties in grammatical structuring, especially in the use of pronouns, plurals, and articles. In terms of receptive language, JR presented difficulties in structuring and functional use. Parental reports reflected even more limited language use in natural contexts, suggesting low generalization of acquired skills in structured situations. The NEUROKID indicated a severe deficit in articulatory language, while performance in visual language was adequate. In addition, he presented a performance well below average in all ITPA language subtests, confirming a profile with severe difficulties in auditory comprehension, syntactic structuring, and verbal production (Table 5).

**Intervention response:** JR participated actively in language-focused tasks, showing some gains in sentence comprehension but persistent expressive difficulties.

CELF-5 results for JR indicated significantly below average performance in all language areas assessed. Percentile ranks below the 10th percentile indicate severe difficulties compared to normative expectations (Table 6).

- **PATIENT 2—SR**

SR showed a mixed linguistic profile. While in structured contexts her performance was acceptable, limitations in spontaneous expression, articulation, and comprehension persisted in natural environments, presenting limited verbal production, with no use of possessive pronouns, adverbs, or temporal concepts. Parents reported a much lower level compared to the clinical language assessment (Table 7).

**Intervention response:** SR showed gradual improvement in structured tasks, with moderate gains in verbal expression and functional use, though spontaneous language remained limited.

CELF-5 results for SR showed variable performance in the language skills of the evaluee. Composite indices indicated higher difficulties in receptive language (4th percentile) and expressive language (7th percentile). Percentiles are compared to normative expectations for age (Table 8).

### 4.4. Differential Diagnosis

- **PATIENT 1—JR**

**With ADHD:** Attentional difficulties and some impulsivities were observed but are part of ASD symptomatology. Formal ADHD testing was not conducted, but the clinical pattern (social reciprocity, behavioral rigidity, and language difficulties) supports ASD as the primary diagnosis.

**With DLD:** JR showed clearly affected expressive language, but his difficulties also extended to receptive language, nonverbal communication, symbolic play, and social interactions. These features are not characteristic of isolated DLD, which allows us to exclude this condition as the main diagnosis.

**With intellectual disability:** The results obtained in the Merrill-Palmer battery indicated cognitive functioning within the average range, which allowed us to rule out the presence of an intellectual disability.

**With epilepsy:** There was no history of epileptic seizures or objective neurological alterations.

- **PATIENT 2—SR**

**With ADHD:** Attention difficulties integrated within ASD clinical profile. ASD features (restricted interests and social rigidity) are predominant. (no formal ADHD test)

**With DLD:** SR presented a clinical profile similar to that of her brother, with severe alterations in social communication, pragmatic language and the presence of restricted interests. These characteristics allow us to rule out DLD as the main diagnosis, given that the absence of social reciprocity and behavioral rigidity are features of ASD, not DLD.

**With intellectual disability:** Cognitive evaluation revealed performance within normal limits, so no intellectual disability criteria were met.

**With epilepsy:** SR has no history of seizures and no compatible neurological findings. No signs suggestive of epilepsy were observed; therefore, this possibility is ruled out.

### 4.5. Analysis of Comorbidities

- **PATIENT 1—JR**

JR presents a complex profile requiring coordinated intervention. Severe food selectivity (milk protein intolerance) leads to frequent vomiting and limited diet. Sensory hypersensitivities, especially olfactory and tactile, may exacerbate these difficulties, influenced by his premature birth and medical history.

Sleep disorders include difficulty initiating sleep, multiple night awakenings, and poor melatonin response, impacting self-regulation and daytime performance. The absence of sphincter control suggests possible maturational delay.

Verbal and working memory difficulties affect academic performance and language learning. Signs compatible with anxiety were observed (based on parental reports), including frequent distress in novel situations and heightened irritability.

- **PATIENT 2—SR**

SR shows moderate comorbidity. She rejects crushed fruits and vegetables but has a more varied diet than her brother. Sensory profile is less altered, with fewer signs of hypersensitivity or avoidance.

Sleep is fragmented (waking to ask for water) and emotional regulation shows signs compatible with social anxiety (parental reports).

Cognitive difficulties in working memory and planning may impact school performance and the organization of daily tasks.

## 5. Discussion

The general aim of this study was to analyze language development in two premature twins with ASD, with special attention provided to their linguistic, communicative, socioemotional, and cognitive profiles, and to relate these findings to the therapeutic strategies applied. Both cases allow for a detailed observation of how prematurity may modulate ASD phenotypes and influence responsiveness to multimodal interventions.

Both twins exhibited global developmental delays consistent with ASD, but their trajectories diverged in expressive and receptive language, executive functioning, and adaptive behavior. These differences likely reflect individual variability within ASD, together with the cumulative neurodevelopmental vulnerability linked to prematurity, which has been associated with atypical white matter connectivity and structural alterations in key language and social brain networks (Rogers et al., 2018; Vandormael et al., 2019).

JR presented a more severe profile, with marked deficits in expressive and receptive language, executive functions, and emotional regulation. His language comprehension in natural contexts was limited and communication remained poorly functional despite average general cognitive abilities. The integration of structured approaches (ABA and TEACCH) provided predictability, reinforced task engagement, and reduced behavioral rigidity, promoting basic communicative exchanges and early autonomy. Clinical observations, session logs, and parent reports indicated functional progress—not necessarily reflected in standardized test scores but evident in qualitative indicators such as increased engagement, improved transitions between tasks, and reduced behavioral rigidity. This functional improvement was also supported by better scores on the BRIEF-2 and ABAS-II scales. At the same time, DIR/Floortime and Hanen interventions promoted affective reciprocity, shared attention, and parent–child interaction quality. The combination of these relational models with structured support led to modest but meaningful improvements in joint attention and emotional engagement, as reported by the parents and therapists. Given that the CELF-5 was administered only post-intervention, improvements in expressive language and communication are interpreted qualitatively—supported by observational and parental data rather than direct pre–post score comparisons.

In SR’s case, cognitive and linguistic skills were closer to age expectations, allowing her to benefit more directly from relational approaches. Post-intervention CELF-5 results indicated relative strengths in syntax and semantics and weaker performance in pragmatics, which aligned with qualitative language samples showing limited generalization of skills to natural contexts. The DIR/Floortime and Hanen—More Than Words programs were central in expanding her communicative flexibility, promoting social initiative, and facilitating spontaneous conversation. Meanwhile, TEACCH and ABA reinforced executive routines, enabling SR to regulate attention and maintain engagement during structured linguistic activities. This complementary interaction between structured and relational components appears critical for consolidating higher-level language functions and pragmatic competence.

Both twins displayed challenges in working memory, planning, and cognitive flexibility, highlighting the vulnerability of executive functions in preterm children with ASD. TEACCH and ABA provided structured routines and reinforcement to support task sequencing and anticipation, while DIR/Floortime maintained motivation and meaningful engagement. Improvements in anticipation and routine consolidation were more evident in SR, whereas JR required consistent support for attention and self-regulation, emphasizing the need for individualized strategies.

Adaptive behavior and personal autonomy, assessed through the ABAS-II, showed limited progress in both twins, with JR remaining at very low levels and SR achieving moderate but below-expected gains. Comorbidities, including sensory hypersensitivity, feeding rigidity, and sleep disturbances, were managed through structured routines and caregiver involvement (Hanen program), highlighting the importance of individualized interventions tailored to both neurodevelopmental and environmental factors.

These findings illustrate how prematurity may exacerbate vulnerabilities associated with ASD, such as attentional regulation deficits, slower processing, and atypical sensory integration, influencing language, cognitive, and socioemotional outcomes. Premature birth interrupts key processes of the brain’s maturation, including myelination and the development of long-range connectivity, which are essential for language and executive functioning (Joseph et al., 2023). As a result, prematurity may amplify difficulties typical of ASD, such as limited flexibility and reduced spontaneous communication (Kalish et al., 2017; Luu et al., 2020). In JR, the compounded effect of prematurity and ASD likely intensified challenges in neural integration, requiring greater environmental structure to achieve basic communicative outcomes. In SR, better cognitive regulation and plasticity allowed her to respond more effectively to relational and pragmatic stimulation. These observations suggest that prematurity interacts with ASD characteristics to shape responsiveness to intervention.

The linguistic results observed in both twins coincide with what was reported by Joseph et al. (2023), who pointed out that children with ASD born prematurely present significant delays in the emergence of vocabulary and sentence construction, in addition to facing more difficulties in organizing discourse and using complex grammatical structures. In the same vein, Harel-Gadassi et al. (2018) found that gestational age is directly related to language performance and social communication, reinforcing the association between prematurity and impairments in language development.

Likewise, the results obtained in the CELF-5 assessment and the pragmatic profile are consistent with the findings of Reidy et al. (2013), who demonstrated that children born very prematurely present significant deficits in phonology, semantics, discourse, and pragmatics at seven years of age. Complementarily, Kalish et al. (2017) and Joseph et al. (2017) evidenced a higher prevalence of ASD in preterm children, as well as a particular phenotype characterized by marked alterations in social communication and a lower presence of repetitive behaviors.

The effectiveness of the therapeutic strategies is supported by the literature: DIR/Floortime improves emotional functioning, communication, daily living skills, and social interaction (Liao et al., 2014); ABA promotes sustained gains in cognitive, linguistic, and social domains (Meçe & Sherifi, 2022); TEACCH enhances expressive language, social reciprocity, affective expression, fine motor skills, and personal autonomy (Zeng et al., 2021); Hanen strengthens caregiver–child interactions, language use, and socioemotional adjustment. These findings demonstrate that multimodal intervention strategies can operate synergistically to address the complex profiles of premature children with ASD.

Among the main limitations is the small sample size, limited to two participants, which restricts generalizability. Furthermore, some standardized tests were only applied post-intervention, which prevents direct quantitative comparison. Nevertheless, qualitative convergence across sources (clinical observation, parent reports, and test results) supports the validity of the interpretations.

Future research should compare premature and term-born children with ASD to determine how gestational age moderates the response to different therapeutic models. A longitudinal perspective would also help us to understand whether gains achieved through multimodal interventions are maintained over time.

## 6. Conclusions

This case report on two premature twins with a diagnosis of ASD provides insight into how the co-occurrence of prematurity and autism spectrum disorder can shape distinct linguistic, cognitive, and adaptive developmental pathways. Although both conditions independently increase the risk of communication and self-regulation difficulties, their combination may amplify these vulnerabilities, resulting in greater heterogeneity in developmental outcomes.

The differentiated profiles observed in JR and SR suggest that some features—such as executive function weaknesses, attentional instability, and slower processing—may be partly related to prematurity, whereas difficulties in social reciprocity, pragmatic language use, and emotional regulation align more closely with core ASD characteristics. This highlights the importance of disentangling overlapping symptoms to design more precise diagnostic and therapeutic approaches for children born preterm who are later diagnosed with ASD.

The findings also demonstrate that early, structured, and sustained multimodal interventions (ABA, TEACCH, DIR/Floortime, and Hanen) can promote meaningful progress in expressive and receptive language, social communication, autonomy, and self-regulation, particularly when the intervention integrates both structured and relational dimensions. Nevertheless, residual challenges in language generalization and pragmatic understanding underline the need for long-term, contextually embedded support.

From a clinical perspective, this case underscores the relevance of individualized assessment frameworks that consider both perinatal history and neurodevelopmental profiles. The combined use of structured teaching and family centered relational approaches proved effective in adapting therapeutic goals to each child’s cognitive and emotional resources. From a research standpoint, this study contributes to the limited literature on intervention outcomes for premature children with ASD, emphasizing the importance of considering prematurity as a potential moderator of therapy responsiveness.

Although the results are not generalizable, this report lays the groundwork for future longitudinal and comparative studies examining differences between preterm and term-born children with ASD, which could clarify how early biological vulnerabilities interact with neurodevelopmental mechanisms. Ultimately, this work advances a more nuanced understanding of how integrated intervention models can support the complex developmental needs of premature children on the autism spectrum.

## Figures and Tables

**Table 1:** Results obtained in the measures of the maturational development of JR.

Area	2022—PT/PC/EE	2024—PT/PC/EE	Interpretation (Post-Intervention)
MP Global Development	96/39/47 months	102/55/51 months	Average
MP Adaptive Behavior	26/2/20 months	40/9/28 months	Very low
ABAS-II Conceptual	-	64/1/-	Very low
ABAS-II Social	-	56/<1/-	Very low
ABAS-II Practical	-	58/<1/-	Very low
ABAS-II Adaptive Behavior	-	57/<1/-	Very low
BRIEF-2 Behavioral Regulation	-	66/2/-	Very low
BRIEF-2 Emotional Regulation	-	71/4/-	Very low
DP-3 Adaptative Behavior	-	112/79/-	High average
DP-3 Socioemotional	-	88/21/-	Low
SENA Behavioral Problems	-	67/4/-	Low
SENA Emotional Problems	-	77/9/-	Low average
SENA Personal Resources	-	15/<1/-	Very low

**Table 2:** Results obtained in the measures of the maturational development of SR.

Area	2022—PT/PC/EE	2024—PT/PC/EE	Interpretation (Post-Intervention)
MP Global Development	101/53/46 months	110/75/54 months	High average
MP Adaptive Behavior	68/25/30 months	80/40/40 months	Low average
ABAS-II Conceptual	-	64/1/-	Very low
ABAS-II Social	-	56/<1/-	Very low
ABAS-II Practical	-	58/<1/-	Very low
ABAS-II Adaptive Behavior	-	57/<1/-	Very low
BRIEF-2 Behavioral Regulation	-	93/32/-	Very low
BRIEF-2 Emotional Regulation	-	99/47/-	Very low
DP-3 Adaptative Behavior	-	50/1/-	Very low
DP-3 Socioemotional	-	50/1/-	Very low
SENA Behavioral Problems	-	69/5/-	Low average
SENA Emotional Problems	-	57/2/-	Very low
SENA Personal Resources	-	25/<1/-	Very low

**Table 3:** Results obtained in the cognitive development measures of JR.

Area	2022—PT/PC/EE	2024—PT/PC/EE	Interpretation (Post-Intervention)
MP Cognition	90/25/43months	95/37/48 months	Average
MP Memory	97/42/46 months	96/46/47 months	Average
MP Speed Processing	91/27/44 months	96/40/47 months	Average
NeuroKid Executive Functions	-	6/37/-	Average
NeuroKid Visuoperception	-	7/50/-	Average
NeuroKid Memory	-	6/37/-	Average
BRIEF-2 Cognitive Regulation	-	77/<1/-	Very low
BRIEF-2 Executive Functions	-	78/<1/-	Very low
DP-3 Cognition		105/63/-	Average
ITPA Auditory Integration	-	65/1/-	Very low
ITPA Visual Association	-	82/12/-	Low
ITPA Visual Memory		75/5/-	Very low
SENA Executive Functions	-	80/9/-	Low average

**Table 4:** Results obtained in the cognitive development measures of SR.

Area	2022—PT/PC/EE	2024—PT/PC/EE	Interpretation (Post-Intervention)
MP Cognition	99/47/44 months	106/66/52 months	High average
MP Memory	99/47/44 months	104/61/50 months	Low average
MP Speed Processing	97/42/43 months	102/55/48 months	Average
NeuroKid Executive functions	-	8/75/-	Average
NeuroKid Visuoperception	-	7/50/-	Average
NeuroKid Memory	-	6/37/-	Average
BRIEF-2 Cognitive Regulation	-	99/50/-	Average
BRIEF-2 Executive Functions	-	99/50/-	Average
DP-3 Cognition		50/1/-	Very low
ITPA Auditory Integration	-	80/10/-	Low
ITPA Visual Association	-	90/25/-	Average
ITPA Visual Memory		95/37/-	Average
SENA Executive Functions	-	92/30/-	Average

**Table 5:** Results obtained in the linguistic development measures of JR.

Area	2022—PT/PC/EE	2024—PT/PC/EE	Interpretation (Post-Intervention)
MP Receptive Language	99//43 months	107/68/53 months	High average
MP Expressive Language (Clinical Eval.)	105//49 months	112/79/56 months	High average
MP Expressive Language (Parents)	20//19 months	50/10/36 months	Low
MP Total Language Index	70//34 months	88/31/44 months	Very low
NeuroKid Articulatory Language	-	1/<1/-	Very low
NeuroKid Visual Language	-	6/37/-	Average
DP-3 Communication		110/75/-	High average
ITPA Auditory Association	-	70/2/-	Very low
ITPA Verbal Expression	-	60/<1/-	Very low
ITPA Grammatical Organization	-	68/2/-	Very low

**Table 6:** Results CELF-5 JR.

Subtest	Scaled Score	Percentile	Performance
Sentence Comprehension	4	2	Very low
Sentence Structure	3	1	Very low
Sentence Formulation	5	5	Low
Related Words	4	2	Very low
Auditory Comprehension	3	1	Very low
Sentence Repetition	2	<1	Very low
Repetition of phrases and pseudowords	3	1	Very low
Index of Receptive Language (IRL)	63	1	Very low
Index of Expressive Language (IEL)	60	<1	Very low
Index of Language Content (ILC)	65	1	Very low
Index of Total Language (ITL)	61	<1	Very low

**Table 7:** Results obtained in the linguistic development measures of SR.

Area	2022—PT/PC/EE	2024—PT/PC/EE	Interpretation (Post-Intervention)
MP Receptive Language	89//42 months	93/32/46 months	Low average
MP Expressive Language (Clinical Eval.)	74//33 months	82/12/40 months	Low
MP Expressive Language (Parents)	19//20 months	35/<1/28 months	Very low
MP Total Language Index	42//32 months	60/<1/38 months	Very low
NeuroKid Articulatory Language	-	4/16/-	Low
NeuroKid Visual Language	-	6/37/-	Average
DP-3 Communication		50/1/-	Very low
ITPA Auditory Association	-	85/16/-	Low average
ITPA Verbal Expression	-	78/7/-	Low
ITPA Grammatical Organization	-	84/14/-	Low

**Table 8:** Results of CELF-5 SR.

**Subtest**	**Scaled Score**	**Percentile**	**Performance**
Sentence Comprehension	7	16	Low average
Sentence Structure	6	9	Low
Sentence Formulation	8	25	Average
Related Words	9	37	Average
Auditory Comprehension	6	9	Low
Sentence Repetition	7	16	Low average
Repetition of phrases and pseudowords	5	5	Low
Index of Receptive Language (IRL)	73	4	Very low
Index of Expressive Language (IEL)	78	7	Low
Index of Language Content (ILC)	82	12	Low
Index of Total Language (ITL)	76	5	Low

## Data Availability

The original contributions presented in this study are included in the article. Further inquiries can be directed to the corresponding authors.
